# Characterization of Vaginal Microbial Colonization in Cervical Insufficiency Patients and Its Relation to Preterm Birth: An Observational Cohort Study

**DOI:** 10.1155/jp/6561798

**Published:** 2025-11-26

**Authors:** Fanny Mikula, Arlena Witt, Ricarda Heemann, Sonja Granser, Florian Heinzl, Alex Farr, Philipp Foessleitner

**Affiliations:** Department of Obstetrics and Gynecology, Division of Obstetrics and Feto-maternal Medicine, Comprehensive Center for Pediatrics, Medical University of Vienna, Vienna, Austria

**Keywords:** cervical incompetence, cervical insufficiency, preterm birth, spontaneous preterm birth, vaginal microbial colonization

## Abstract

**Background:**

Cervical insufficiency is one of the main risk factors for preterm birth. It has been suggested that a more diverse vaginal microbial colonization might lead to cervical insufficiency and subsequently further increase the risk for preterm birth. To date, the microbial colonization in women with cervical insufficiency has not been sufficiently categorized. Therefore, this study is aimed at describing the vaginal microbial colonization in this high-risk collective and exploring a possible association with preterm birth.

**Methods and Study Design:**

All women treated for cervical insufficiency from June 2021 until March 2024 at the Division for Obstetrics and Feto-Maternal Medicine of the Medical University of Vienna were evaluated for inclusion. Vaginal bacterial/fungal culture results during pregnancy were used for the characterization of the vaginal microbial colonization and categorized in 17 predefined microbial groups.

**Results:**

We included 118 women with cervical insufficiency with available vaginal culture results, of whom 58.5% experienced preterm birth. *Lactobacillus* spp., coagulase-negative staphylococci, *Enterococcus* spp. and *Ureaplasma* spp. were the most frequently detected microorganisms. Further, we conducted a secondary exploratory analysis of the association of each individual microbial group with preterm birth, which found an absence of lactobacilli (*p* = 0.047) and the presence of a more diverse microbial composition with Gram-negative anaerobes, *Ureaplasma* spp. and *Enterococcus* spp. to be more frequent in PTB.

**Conclusion:**

Cervical insufficiency is associated with a diverse vaginal microbial colonization. Especially colonization with coagulase-negative staphylococci, *Ureaplasma* spp., and *Enterococcus* spp. seems to play an important role in cervical insufficiency. *Lactobacillus* spp. absence was associated with subsequent preterm birth.

## 1. Introduction

Cervical insufficiency (CI) is one of the leading risk factors for preterm birth (PTB), defined as delivery before 37 gestational weeks [1, 2]. According to the World Health Organization, approximately 13 million babies are born prematurely every year, with PTB representing the leading cause of child mortality worldwide [3]. Infants born extremely preterm, defined as delivery before 28 weeks of gestation, face the highest risk of adverse outcomes, including perinatal mortality [3]. CI is defined by a shortened cervix typically during the second trimester of pregnancy. Although varying cut-off values are described, the most commonly used definition is a cervical length ≤ 25 mm before 34 gestational weeks [4, 5]. The main challenge in the management of CI is to prevent PTB. Possible measures include vaginal or oral progesterone, tocolysis, cervical cerclage, or pessaries, although some of these options are controversial [4–9].

The vaginal detection of immunological markers, such as interleukin-8 or E-selectin, has been identified as a potential predictive indicator for imminent PTB in women with CI [10–12]. Additionally, elevated maternal serum inflammatory parameters appear to be associated with an increased risk for PTB in this patient cohort [13]. These findings support the hypothesis that conditions that induce a local inflammatory reaction, such as a diverse and potentially pathogenic vaginal microbial colonization, might promote PTB in women suffering from CI. Multiple studies have found an association between a more diverse vaginal microbiota and spontaneous PTB as well as its associated complications [14–17]. Steetskamp et al. [16] conducted a characterization of the vaginal bacterial colonization in pregnant women with asymptomatic short cervix and found a predominance of *Ureaplasma* species (spp.) and *Escherichia* (*E.*) *coli*. Nevertheless, this retrospective study found no correlation between bacterial colonization and PTB and suggested that further research should explore the clinical implications of these microbial findings [16]. Therefore, the aim of this study was to further characterize the vaginal microbial colonization in women with CI and to investigate whether the microbiological colonization correlates with the risk of PTB.

## 2. Methods

### 2.1. Aims and Endpoints

The primary endpoint of the presented study was to examine and characterize the microbial colonization in women suffering from CI. The secondary endpoint was the evaluation of a potential correlation between the presence of certain microbial groups and the risk for preterm delivery on an exploratory basis.

### 2.2. Study Design and Patients

We performed a retrospective observational cohort study. It received approval from the ethical committee of the Medical University of Vienna (Application Number: 1342/2024) and was conducted in accordance with the Declaration of Helsinki and Good Scientific Practice guidelines as well as the STROBE checklist for cohort studies [18]. Pregnant women treated for CI at the Division for Obstetrics and Feto-Maternal Medicine of the Medical University of Vienna from June 2021 until March 2024 were evaluated for inclusion. CI was diagnosed through vaginal sonography, performed in accordance with locally applicable guidelines [4]. The procedure was conducted with an empty bladder, ensuring that the entire longitudinal axis of the cervix occupied approximately 50%–75% of the ultrasound screen. Care was taken to avoid applying pressure on the cervix with the ultrasound probe. A minimum of three measurements were obtained, with the shortest measurement being used to guide clinical management. CI was diagnosed when the cervical length was measured at ≤ 25 mm before 34 + 0 weeks of gestation [4]. At our department, vaginal swabs are routinely collected from women diagnosed with CI for various diagnostic purposes, including microscopy, Gram staining, bacterial and fungal cultures, and molecular testing for specific microorganisms such as *Ureaplasma* spp. and *Chlamydia trachomatis.* Swabs are collected during speculum examination from the lateral vaginal wall and posterior fornix. The following women were excluded from our study: (i) multiple pregnancies, (ii) age < 18 or > 55 years, (iii) cervical cerclage in ongoing pregnancy, (iv) history of cervical surgery, (v) missing culture/molecular specimens within 1 week of CI diagnosis, and (vi) iatrogenic PTB.

### 2.3. Clinical Management and Data Collection

A vaginal swab for culture and molecular analysis was routinely collected following CI diagnosis. The detected microbes were divided into 17 predefined microbial groups according to microbiological features as well as clinical relevance. The 17 groups are listed in Table 1. *Chlamydia trachomatis* was not included in the predefined microbial groups, because no participant tested positive. The management of CI at our tertiary care center is based on a comprehensive, multidisciplinary approach. Antibiotic and antifungal treatments as well as probiotic treatment containing lactobacilli were administered when indicated, guided by Gram smear, culture, and molecular test results. Bacterial vaginosis, diagnosed using Nugent scoring, was treated with local or systemic clindamycin. Vulvovaginal candidosis, diagnosed by the detection of (pseudo-)hyphae, was treated with local clotrimazole or oral fluconazole. Detection of *Ureaplasma* spp. in high-risk pregnancies routinely warranted a single-dose azithromycin treatment. Vaginal progesterone is routinely prescribed to women with CI, provided no contraindications are present. In cases of premature contractions or progression of CI, tocolytic therapy is implemented. For women with a high estimated risk of PTB, antenatal corticosteroids are offered for respiratory distress syndrome prophylaxis before 34 weeks of gestation. Additionally, magnesium sulfate is administered for neuroprotection in cases of imminent PTB before 32 weeks of gestation.

Demographic and perinatal data were collected using the obstetric documentation system ViewPoint Fetal Database, Version 5.6.28.56 (General Electric Company, GE Viewpoint, Munich, Germany) and the hospital information system (SAP SE Inc., Walldorf, Germany). Data collection was monitored by two separate investigators to ensure accurate data quality.

### 2.4. Statistical Analysis

First, we conducted a descriptive analysis of maternal parameters. Categorical variables were summarized as absolute and relative frequencies. Continuous variables were described using median and interquartile range (IQR) for nonnormally distributed data and mean ± standard deviation (SD) for normally distributed data. For the primary endpoint, microbial colonization was analyzed as a binary variable, with microbial groups classified as either present or absent. For the secondary endpoint, potential associations between the presence of specific microbial groups and the risk of PTB were assessed using chi-square tests. It is important to emphasize that this analysis is strictly exploratory in nature; as such, *p* values are not intended to confirm statistical significance but rather to identify potential trends for further investigation. Statistical calculations were performed using R (R Core Team 2024, Vienna, Austria) [19–25].

## 3. Results

### 3.1. Patient Collective

We included 118 participants with CI who met the inclusion criteria. The median gestational age at diagnosis of CI was 25 + 4 (IQR, 23 + 2–29 + 2) gestational weeks with a median cervical length of 17 mm (11.8–22). At the time of CI diagnosis, 35 participants (29.7%) suffered from an active vulvovaginal infection, either bacterial vaginosis or vulvovaginal candidosis. The median gestational age at delivery was 35 + 6 (31 + 3–38 + 6) weeks with a mean birthweight of 2396 g (SD, ± 1062 g). In total, 69 of 118 (58.5%) participants delivered before 37 + 0 gestational weeks and were therefore categorized as PTB. The maternal characteristics and neonatal outcomes are summarized in Table 2.

### 3.2. Microbial Colonization

As outlined in the Methods section, we predefined 17 microbial groups and determined the presence of each group for every participant. The most frequently detected microbial group was *Lactobacillus* spp. (*n* = 93/118; 78.8%). Other commonly observed microbes included coagulase-negative staphylococci (*n* = 87/118; 73.7%), *Ureaplasma* spp. (*n* = 46/118; 39%), *Enterococcus* spp. (*n* = 46/118; 39%), and Gram-negative anaerobes (*n* = 39/118; 33.1%). All predefined microbial groups were detected within the cohort, but some were rare, such as *Staphylococcus aureus* (*n* = 2/118; 1.7%) and other beta-hemolytic streptococci (*n* = 2/118; 1.7%). A detailed overview of microbial findings is provided in Table 3.

### 3.3. PTB

**Figure 1:**
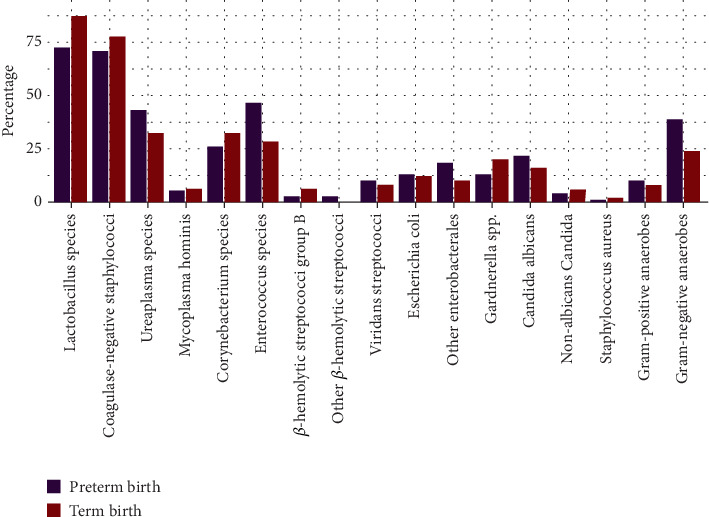
The relative frequency of detection of each microbial group in participants with subsequent term birth (red) and preterm birth (violet).

As a secondary endpoint, we conducted an exploratory analysis to assess potential associations between microbial colonization in pregnant women with CI and their risk of PTB. The results are presented in Figure 1. The most notable difference was observed in the presence of *Lactobacillus* spp., which were detected in 43/49 (87.8%) women with term delivery but only in 50/69 (72.5%) women with preterm delivery (*p* = 0.047). Other microbial groups, such as Gram-negative anaerobes (24.5% vs. 39.1%; *p* = 0.108), *Enterococcus* spp. (28.6% vs. 46.4%; *p* = 0.052), and *Ureaplasma* spp. (32.7% vs. 43.5%; *p* = 0.341), were more frequently detected in the PTB cohort.

In total, nine microbial groups were more common in the PTB cohort compared to the term birth cohort, including *viridans* streptococci, *E. coli*, other enterobacterales, *Candida albicans*, as well as both Gram-negative and Gram-positive anaerobes. Notably, other beta-hemolytic streptococci were found exclusively in the PTB cohort, although this group was rare overall, with positive results in only two participants (1.7%).

The detailed findings for each microbial group are provided in Table 4. It is important to note that due to the multiple statistical tests conducted, this analysis is exploratory and not designed to establish statistical significance, but rather to identify potential trends for further investigation.

## 4. Discussion

CI is a significant risk factor for PTB [26]. In our study cohort, consisting exclusively of women with CI, 58.5% of pregnancies resulted in PTB. Vaginal microbial colonization in this cohort was diverse, with *Lactobacillus* spp., coagulase-negative staphylococci, *Ureaplasma* spp.*, Enterococcus* spp., and Gram-negative anaerobes being the most frequently detected microorganisms. Notably, we observed distinct differences in the microbial communities between women with subsequent PTB compared to those with term birth, including an absence of lactobacilli and increased microbial diversity in the PTB group.

Although the exact etiology of spontaneous PTB remains not fully understood, inflammatory pathways appear to play a significant role [27]. In women diagnosed with CI, studies have shown that the presence of inflammatory biomarkers in vaginal or cervical swabs may help predict PTB [10, 11]. These findings suggest that the vaginal microbial colonization could influence the risk of PTB. This hypothesis has already been well established for uncomplicated pregnancies [28, 29]. Gudnadottir et al. [28] and Zhou et al. [29] showed in their respective meta-analyses that a more diverse vaginal microbial composition with a low abundance of lactobacilli was significantly associated with a higher risk for PTB. Similarly, studies have also explored vaginal microbial colonization in women with CI [16, 30, 31]. Choi et al. [30] reported a high prevalence of *E. coli* in this population, with 33% of women testing positive for this bacterium. In contrast, our analysis found a lower prevalence, with *E. coli* detected in only 13% of participants' vaginal swabs. Steetskamp et al. [16] recently reported that the vaginal microbial colonization in women with CI is highly diverse, with only 34% of women in their cohort exhibiting normal vaginal microbiota. The most frequently detected pathogens in their cohort were *Ureaplasma* spp., which were found in approximately 30% of participants [16], consistent with our findings showing a 39% colonization rate. Silvano et al. [31] further supported the observation that women with CI tend to have more diverse microbial colonization. Their study demonstrated significantly enriched bacterial taxa, including an overrepresentation of microbes such as staphylococci and *Gardnerella vaginalis*, in women with a short cervix, compared to those with a normal cervical length [31].

In our high-risk cohort for PTB, lactobacilli were detected in 79% of women, whereas Dutt et al. reported a higher prevalence of 88% in low-risk pregnant Caucasian women [32]. This observation supports the hypothesis that the presence of *Lactobacillus* spp. exerts a protective effect against PTB, particularly considering that *Lactobacillus* spp. were identified in only 73% of participants in our study who experienced PTB. In contrast, Gram-negative bacteria [33] and Gram-negative anaerobes [32] have been reported in 10% and 8% of low-risk pregnant women, respectively, whereas Gram-negative anaerobes were identified in 33% of women in our high-risk cohort, and in as many as 39% of those who experienced a PTB. This suggests that the presence of Gram-negative anaerobes may be associated with an increased risk of PTB. Together, these findings highlight the importance of microbial balance—particularly the predominance of *Lactobacillus* spp. and the relative absence of Gram-negative anaerobes—in maintaining a healthy vaginal milieu during pregnancy. Interestingly, compared with low-risk pregnancies (55%–67%) [32, 33], *Ureaplasma* spp. were detected less frequently in our high-risk cohort (39%), a rate similar to that reported in other CI populations as published by Steetskamp et al. [16]. Nevertheless, *Ureaplasma* spp. were considerably more common among women in our cohort who experienced PTB compared with those who delivered at term (44% vs. 33%). These findings underscore the complexity of the still unresolved association between *Ureaplasma* spp. colonization and PTB risk.

In line with the summarized evidence, our data suggest that increased microbial diversity, especially regarding Gram-negative anaerobes, coupled with the absence of lactobacilli, appears to be a causative factor in CI. Probiotics containing *Lactobacillus* spp. have been established as a potential treatment or prophylaxis for various conditions characterized by similarly increased bacterial diversity, such as chronic vaginitis [34, 35]. A beneficial effect of probiotic treatment has also been observed in pregnant women with intermediate bacterial microbiota as it increases gestational age at delivery and, consecutively, the birth weight [36, 37]. To the best of our knowledge, no study has yet investigated the effects of *Lactobacillus*-containing probiotics in women with CI. Our findings suggest that testing such an intervention could be beneficial for women affected by CI.

Regarding the risk of subsequent PTB, current evidence suggests that vaginal colonization with certain pathogens may further increase the risk in women with CI. Bacteria such as *Ureaplasma* spp. and *E. coli*, as well as the absence of lactobacilli, have been associated with an elevated PTB risk in this high-risk population [16, 30]. However, findings across studies are heterogeneous, and there is consensus that further research is needed to clarify these associations [16, 28–30]. Our study offers exploratory analyses of potential associations between colonization by specific microbial groups and the risk of subsequent PTB following CI. Consistent with previous findings, our data suggest that lactobacilli abundance may have a protective effect against PTB [29]. Certain bacteria, most notably Gram-negative anaerobes and *Ureaplasma* spp., were detected more frequently in the PTB cohort, indicating a possible correlation. In contrast, other bacteria, such as *E. coli*, showed similar colonization rates in participants with term and PTBs.

We acknowledge the limitations of our study. First, as a retrospective study, it is susceptible to selection bias. While our findings provide valuable insights into microbial colonization in the high-risk cohort of pregnant women with CI, we emphasize that our analyses were exploratory in nature, aiming to evaluate the association between individual microbial groups and the risk of PTB. As such, these data do not permit definitive conclusions but rather serve as a foundation for further, more targeted research. Moreover, our study exclusively examines the increased risk of PTB in a CI cohort following adequate treatment guided by Gram smear, culture, and molecular analyses, including treatment of bacterial vaginosis, vulvovaginal candidosis, or colonization with *Ureaplasma* spp. It is likely that microbial colonization could exert a more significant effect on PTB risk in cases where routine diagnostic measures and subsequent adequate treatment are not administered. However, we strongly believe that withholding treatment for vaginal infections or potentially pathogenic microbial colonization in a high-risk population would not be ethically justifiable.

## 5. Conclusion

Our study identified a diverse microbial colonization in women with CI, with *Lactobacillus* spp., coagulase-negative staphylococci, *Enterococcus* spp., and *Ureaplasma* spp. being the most frequently detected microbial groups. In our exploratory analysis, we observed that the absence of *Lactobacillus* spp. and a more diverse vaginal colonization were associated with an increased risk of PTB. However, given the exploratory nature of our findings, further research is needed to precisely determine the impact of these microbial groups on PTB risk and evaluate potential treatment options.

## Figures and Tables

**Table 1:** The 17 predefined microbial groups for the analysis of the vaginal microbial colonization in patients with confirmed cervical insufficiency.

**Microbial groups**
*Lactobacillus* species
Coagulase-negative staphylococci
*Ureaplasma* species
*Mycoplasma hominis*
*Corynebacterium* species
*Enterococcus* species
Beta-hemolytic streptococci group B
Other beta-hemolytic streptococci
*Viridans* streptococci
*Escherichia coli*
Other enterobacterales
*Gardnerella vaginalis*
*Candida albicans*
Non-albicans *Candida*
*Staphylococcus aureus*
Gram-positive anaerobes
Gram-negative anaerobes

**Table 2:** Maternal characteristics and perinatal outcome parameters of the study cohort.

**Characteristic**	**Participants (** **n** = 118**)**
Maternal age in years (median + IQR)	32 (28–36)
Gravidity (median + IQR)	2 (1–3)
Parity (median + IQR)	0 (0–1)
BMI (median + IQR)	24.4 (21–28.9)
Cervical length at diagnosis in mm (median + IQR)	17 (11.8–22)
Gestational age at diagnosis in weeks (median + IQR)	25 + 4 (23 + 2–29 + 2)
Gestational age at delivery in weeks (median + IQR)	35 + 6 (31 + 3–38 + 6)
PTB (absolute + relative frequency)	69 (58.5%)
Extreme PTB (absolute + relative frequency in the PTB cohort)	20 (29%)
Very PTB (absolute + relative frequency in the PTB cohort)	12 (17.4%)
Moderate-to-late PTB (absolute + relative frequency in the PTB cohort)	37 (53.6%)
Birth weight in grams (mean ± SD)	2396 ± 1062
Umbilical pH value (median + IQR)	7.28 (7.23–7.34)
5-min Apgar (median + IQR)	9 (9–10)
Neonatal infection (absolute + relative frequency)	8 (6.8%)

*Note:* Extreme PTB, delivery before 28 + 0 weeks; very PTB, delivery before 32 + 0 weeks; moderate-to-late PTB, delivery before 37 + 0 weeks.

Abbreviations: BMI, body mass index; IQR, interquartile range; SD, standard deviation.

**Table 3:** Vaginal microbial colonization in the study cohort with cervical insufficiency, categorized into 17 predefined microbial groups and their presence described using absolute and relative frequencies.

**Microbial group**	**Detectable colonization (** **n** = 118**)** **Absolute and relative frequency**
*Lactobacillus* species	93 (78.8%)
Coagulase-negative staphylococci	87 (73.7%)
*Ureaplasma* species	46 (39%)
*Mycoplasma hominis*	7 (5.9%)
*Corynebacterium* species	34 (28.8%)
*Enterococcus* species	46 (39%)
Beta-hemolytic streptococci group B	5 (4.2%)
Other beta-hemolytic streptococci	2 (1.7%)
*Viridans* streptococci	11 (9.3%)
*Escherichia coli*	15 (12.7%)
Other enterobacterales	18 (15.3%)
*Gardnerella vaginalis*	19 (16.1%)
*Candida albicans*	23 (19.5%)
Non-albicans *Candida*	6 (5.1%)
*Staphylococcus aureus*	2 (1.7%)
Gram-positive anaerobes	11 (9.3%)
Gram-negative anaerobes	39 (33.1%)

**Table 4:** Microbial colonization in the cervical insufficiency cohort, stratified by the occurrence of preterm birth, showing the relative frequencies of 17 defined microbial groups in term and preterm birth groups, along with their corresponding exploratory *p* values.

**Microbial group**	**Detectable colonization (** **n** = 118**)**		
	**Term birth, ** **n** = 49**(n, %)**	**Preterm birth, ** **n** = 69**(n, %)**	**p** ** value**
*Lactobacillus* species	43 (87.8%)	50 (72.5%)	0.047
Coagulase-negative staphylococci	38 (77.6%)	49 (71%)	0.571
*Ureaplasma* species	16 (32.7%)	30 (43.5%)	0.341
*Mycoplasma hominis*	3 (6.1%)	4 (5.8%)	0.961
*Corynebacterium* species	16 (32.7%)	18 (26.1%)	0.571
*Enterococcus* species	14 (28.6%)	32 (46.4%)	0.052
Beta-hemolytic streptococci group B	3 (6.1%)	2 (2.9%)	0.471
Other beta-hemolytic streptococci	0 (0%)	2 (2.9%)	0.283
*Viridans* streptococci	4 (8.2%)	7 (10.1%)	0.740
*Escherichia coli*	6 (12.2%)	9 (13%)	0.914
Other enterobacterales	5 (10.2%)	13 (18.8%)	0.206
*Gardnerella vaginalis*	10 (20.4%)	9 (13%)	0.341
*Candida albicans*	8 (16.3%)	15 (21.7%)	0.571
Non-albicans *Candida*	3 (6.1%)	3 (4.4%)	0.688
*Staphylococcus aureus*	1 (2%)	1 (1.5%)	0.908
Gram-positive anaerobes	4 (8.2%)	7 (10.1%)	0.740
Gram-negative anaerobes	12 (24.5%)	27 (39.1%)	0.108

## Data Availability

The data that support the findings of this study are available from the corresponding author upon reasonable request.

## Nomenclature

CI: cervical insufficiency
E: *Escherichia*
IQR: interquartile range
PTB: preterm birth
spp.: species
